# Rapid Identification of Antioxidant Compounds of *Genista saharae* Coss. & Dur. by Combination of DPPH Scavenging Assay and HPTLC-MS

**DOI:** 10.3390/molecules19044369

**Published:** 2014-04-09

**Authors:** Djamila Meriane, Grégory Genta-Jouve, Mohamed Kaabeche, Sylvie Michel, Sabrina Boutefnouchet

**Affiliations:** 1 Laboratoire de Phytothérapie Appliquée aux Maladies Chroniques, Université Ferhat Abbas, Sétif 19000, Algeria; E-Mails: merianed@yahoo.fr (D.M.); Labo_brpdz19@hotmail.com (M.K.); 2 Laboratoire de Pharmacognosie, U.M.R./C.N.R.S. 8638, Faculté des Sciences Pharmaceutiques et Biologiques, Université Paris Descartes, Sorbonne Paris Cité, 4 Avenue de l’Observatoire, F-75006 Paris, France; E-Mails: gregory.genta-jouve@parisdescartes.fr (G.G.-J.); sylvie.michel@parisdescartes.fr (S.M.)

**Keywords:** antioxidant, HPTLC-MS, *Genista saharae*, DPPH scavenging assay

## Abstract

*Genista* species are sources of antioxidant phenolic compounds such as *O*- and *C*-glycosylflavonoids and isoflavonoids. A combination of a DPPH scavenging assay with HPTLC-MS, a fast and efficient method for identification of bioactive compounds, has been applied for evaluation of the radical scavenging activity of metabolites from *Genista saharae* Coss. & Dur. Different organs collected at various periods have been compared. Identification of antioxidant compounds was obtained by elution of the major DPPH-inhibition zones. The resulting HPTLC-MS analysis under moderately polar conditions, coupled to the DPPH results led to the putative identification of two antioxidant isoflavone aglycones: 3',4',5,7-tetrahydroxyisoflavone (**1**) and ficuisoflavone (**3**), whereas polar migration conditions led to the identification of the glycosides 5-methoxy-4',7-trihydroxy-8-glucopyranosylisoflavone (**4**) and 4',5-dihydroxy-7-methoxyisoflavone-4'-*O*-β-d-gluco-pyranoside (**5**). Evaluation of percentage of inhibition of DPPH radical by the purified isoflavone **4** from the root extract showed that it affords a moderate contribution to the total radical scavenging activity of the extract.

## 1. Introduction

Oxidative stress is implied in many inflammatory processes related to chronic inflammatory diseases such as cardiac dysfunction, neurodegenerative diseases or diabetes [1]. A large number of phenolic compounds such as flavonoids, isoflavones, and phenolic acids have shown antioxidant activity. Benefits of antioxidant phenolic compounds from dietary or food supplement sources in the prevention of chronic diseases have been reported in a large number of studies [2,3]. The *Genista* genus, from the family of Fabaceae, consists in 87 species, mainly represented in the Mediterranean area. Among them, 11 species are endemic of Algeria [4]. Phytochemicals isolated from *Genista* species consist in piperidinic alkaloids, *O*- and *C*-glycosylflavonoids and isoflavonoids [5]. *Genista* species have various uses through Mediterranean area, such as dietary or medicinal applications, animal feeding, and also tinctorial use. Antioxidant activities of several *Genista* species have been reported in the literature for crude extracts of *G. tenera, G. sessifolia and G. tinctoria, G. cadasonenesis, G. sandrasica and G. vuralii.* [6,7,8,9]. *Genista saharae* Coss. & Dur. Section *Spartidium* Spach. (formerly *Spartidium saharae* Coss. & Dur) is a Saharian endemic shrub, localised in North Africa (Algeria, Libya, Morocco, Tunisia, Egypt) [4,10]. It is mainly used for feeding animals.

## 2. Results and Discussion

Different samples of *Genista saharae* were collected at the station of Chott el Hodna (Oued El-Maadher, Boussaâda, Wilaya of M’Sila, Algeria) in relation with different phenologic states of the plants, corresponding to the flowering phase and fructification phase. Stems, flowers and roots have been collected. Before DPPH scavenging assay and HPTLC-MS analysis, a preliminary evaluation of total phenolic and flavonoids content of the different organs of collected sample was performed (Table 1). Flavonoids content vary from 0.212% to 2.614%, and total phenolic content from 1.996% to 9.328%. Highest amounts for both were observed for roots collected in August and flowers collected in March. Regarding flowers, these results were in accordance with the ecophysiology of phenolic biosynthesis related to flower development, in which the transcription rate of biosynthetic genes is enhanced [11]. Concerning the roots, it is well documented that plants from the Fabaceae family produce flavonoids in the rhizosphere to stimulate nodulation factors (Nods) [12]. Interestingly, we observed that the flavonoids amount was 10-fold higher when roots were collected in August (2.441%) *versus* in March (0.221%), which is in favor of an important nodulation process during this period. 

In order to screen our extracts for antioxidant compounds we used a DPPH scavenging assay as previously described [13,14]. The different organs of the plant were extracted following the literature protocol and 2 µg of each sample were been applied on the plate using an automated sampler. The first plate was eluted with a 95:5 v/v mixture of dichloromethane and methanol using the automated development chamber and a 5 min solvent pre-saturation. Following the elution, the plate has been dried for 5 min.

**Table 1:** Measured phenolic and flavonoid content of each extract.

Sample	Part	Harvesting period	Extraction Yield (%)	% Flavonoids content (SD)	% Total phenolic content (SD)
**1**	Roots	August 2009	16.31	2,441(0,012)	9,328(0,217)
**2**	Roots	March 2010	8.78	0,212(0,014)	5,490(0,198)
**3**	Stems	August 2009	10.93	0,213(0,022)	5,799(0,148)
**4**	Stems	March 2010	15.07	0,924(0,012)	6,809(0,106)
**5**	Flowers	March 2010	18.00	2,614(0,028)	9,067(0,144)
**6**	Fruit pods	August 2009	6.69	1,273(0,012)	5,062(0,163)
**7**	Fruit Seeds	August 2009	7.70	0,518(0,016)	1,996(0,163)

**Figure 1:**
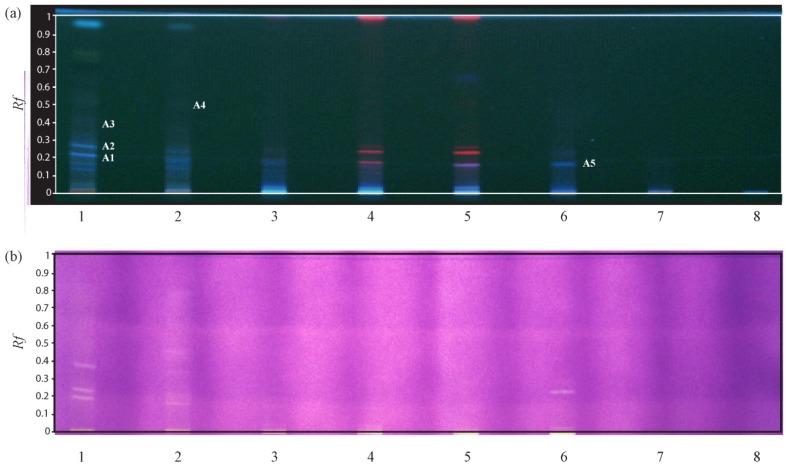
HPTLC analysis, tracks 1–7: *G. saharae* methanolic extracts of samples 1–7. Track 8: purified 5-methoxy-4',7-trihydroxy-8-glucopyranosylisoflavone (**a**) HPTLC detection at 366 nm; (**b**) Picture of the plate after reaction with DPPH.

A first observation of the plate under UV at 366 nm led to the conclusion that an important part of the extract was not eluted using this mobile phase leaving an important spot at *R_f_* = 0 for almost all the tracks. Nonetheless, several compounds were separated under these conditions, especially in tracks 1, 4 and 5 (Figure 1). We further proceed with the antioxidant activity assay by spraying a 0.05% solution of 2,2-diphenyl-1-picrylhydrazyl (DPPH) on the plate. Observation of the plate evidenced several yellow bands indicating the locations of antioxidant compounds (Figure 1).

Five spots were identified as putative moderate antioxidant compounds and in order to proceed with the identification of the compounds, the TLC-MS interface was used on a second plate (using exactly the same protocol). Acquisition of the mass spectra was realised in both positive and negative ionisation modes giving better results in the negative one. The most probable *m*/*z* values for each spot are given in Table 2. The *m*/*z* values were then compared to the data contained in the dictionary of natural products (DNP) for the genus *Genista* [15]. From the several ions observed on the MS spectrum of the spot A1, the most intense corresponded to the *m/z* 299.2 [M−H]^−^.

**Table 2:** Most probable *m*/*z* value and putative structure for each spot.

Spot numbers	*m*/*z* values [M−H]^−^	Putative structures
A1	299.2	-
A2	285.1	3',4',5,7-Tetrahydroxyisoflavone (**1**)
A3	351.2	Hydroxyalpinumisoflavone (**2**)
A4	465.2	-
A5	285.1/353.1	Tetrahydroxyisoflavone (**1**)/ficuisoflavone (**3**)
B1	-	-
B2	445.2	5-Methoxy-4',7-trihydroxy-8-glucopyranosyl (**4**)
B3	445.2	4',5-Dihydroxy-7-methoxyisoflavone-4'-*O*-β-d-glucopyranoside (**5**)
B4	283.1	4',5-Dihydroxy-7-methoxyisoflavone (**6**)
B5	445.2/575.6/325.2	-

The database query for this value did not give any hits and in order to obtain some information on the compound family, a third plate was realised and further derivatised using 2-aminoethyldiphenyl borate. Unfortunately, this operation brought out a very high number of bands (implying a very large number of compounds) but did not give a clear evidence to conclude the family of the compound present in spot A1.

**Figure 2:**
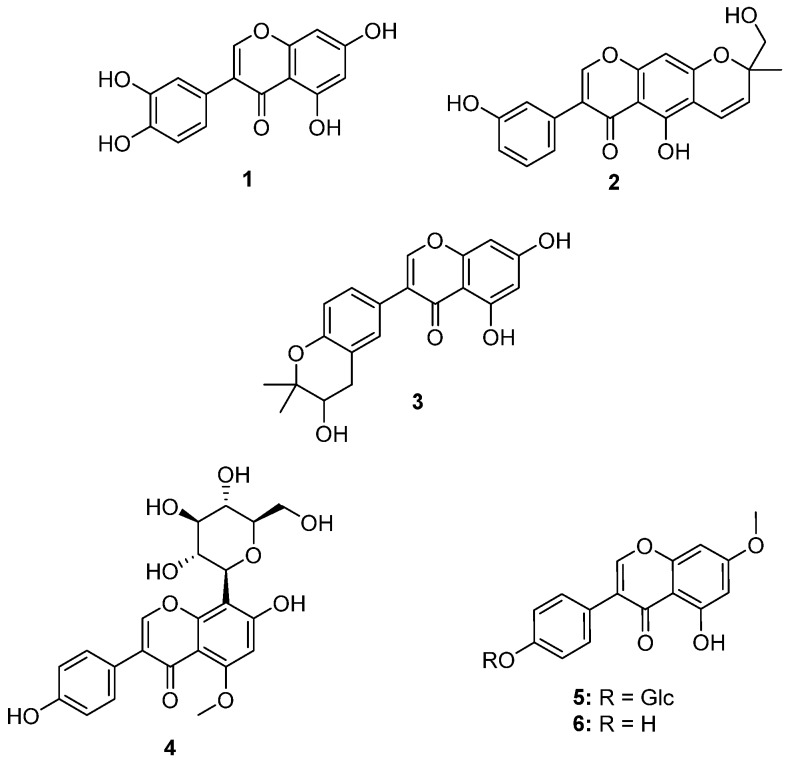
Putative structures of identified compounds.

The analysis was followed by the analysis of spot 2. The mass spectrum obtained gave a one major ion at *m*/*z* 285.1 [M–H]^−^. Although no compound corresponding to this molecular weight was found in the output from the DNP, based on the molecular weight and the retention factor (*R_f_*), this compound was proposed to be a tetrahydroxyisoflavone. Only one tetrahydroxyisoflavone has been reported from plant of the *Genista* genus, the compactin isolated from *Genista compacta* [16]. As compactin is glycosylated, the product found in spot A2 could be the corresponding aglycone, *i.e.*, 3',4',5,7-tetrahydroxyisoflavone (**1**). The MS analysis of the third spot gave also one major ion at *m*/*z* 351.2 and a database search for the genus *Genista* gave only one output: hydroxyalpinumisoflavone (**2**), previously reported from *Genista ephedroides* [17]. Despite the nice isotopic peak observed in the mass spectrum of A4 at *m*/*z* 465.2, no conclusion could be made on the identity of this compound. The last spot (A5) that has been studied using the MS was located in track 6 corresponding to the seeds extract gave a mixture of two ions at *m*/*z* 285.1 (80%) and 353.2 (20%). The first ion was already observed in the first extract and thus could be putatively identified as **1**. The second important ion was observed at *m*/*z* 353.2 [M–H]^−^, and query of the DNP for this target mass in this genus gave only one output: ficuisoflavone (**3**), previously isolated from *Genista corsica* [18]. The structures of these compounds are shown in Figure 2.

**Figure 3:**
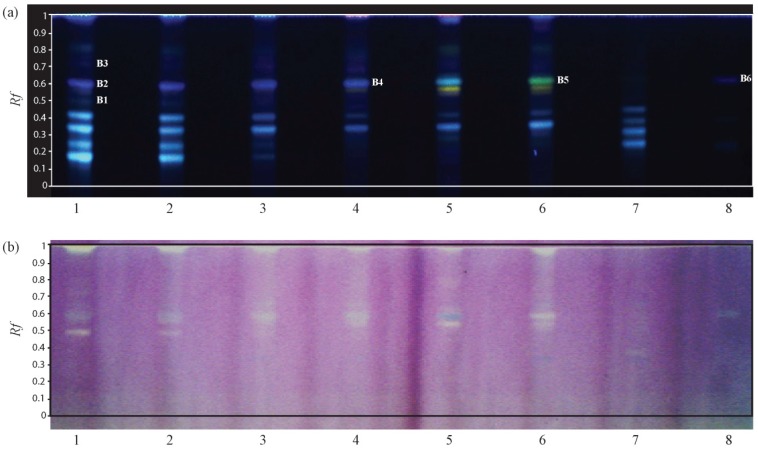
HPTLC analysis, tracks 1–7: *G. saharae* methanolic extracts of samples 1–7. Track 8: purified 5-methoxy-4',7-trihydroxy-8-glucopyranosylisoflavone (**a**) HPTLC detection at 366 nm after derivatisation with 2-aminoethyldiphenyl borate; (**b**) Picture of the plate after reaction with DPPH.

Although several compounds separated using the 95:5 v/v mixture of dichloromethane and methanol exhibited antioxidant activity, an important yellow spot was observed for almost all tracks at *R_f_* = 0. This indicated that a larger number of compounds reacting with DPPH could be separated using a more polar eluant. A mixture, typically used for flavonoids [19], namely ethyl acetate, formic acid, acetic acid, water 100:11:11:26 v/v/v/v was then used for the development of the plates. The change of eluant resulted in a nice separation of other antioxidant compounds as shown in Figure 3.

As illustrated in Figure 3, all the different extracts had compounds reacting with DPPH. Six spots were identified and further studied by HPTLC-MS using the previously described conditions. The identification started with the first spot of the track 1 (B1). Despite a clear antioxidant activity, the acquisition of the mass spectrum of the compound did not lead to clear ions that could be identified. Better results were obtained with the elution of the spot B2 using the HPTLC-MS interface. Only one important ion at *m*/*z* 445.2 was observed and looking at the DNP, two glycosylated isoflavones were consistent with this molecular weight: 5-methoxy-4',7-trihydroxy-8-glucopyranosylisoflavone (**4**) and 4',5-dihydroxy-7-methoxyisoflavone-4'-*O*-β-d-glucopyranoside (**5**), the first being isolated from *Genista saharae* [20]. Interestingly, the third spot of the track 1 (B3) gave the same result during the MS analysis. As already observed for B2, only one intense ion was present on the mass spectrum at *m*/*z* 445.2 [M–H]^−^. In order discriminate those two compounds, an evaluation of the theoretical logP values was realised using the ChemBioDraw Ultra 13.0 Suite (PerkinElmer Inc., Waltham, MA, USA). The compound eluted from spot B2 should have a logP value lower than the one present in B3 due to its reduced migration distance on the plate. The values calculated were ‒0.62 and 0.17 for **4** and **5**, respectively. These results led to the attribution of the structure to each spot as follows: **4** was located in spot B2 and **5** was located in spot B3. The next spot that has been studied was located in track 4 corresponding to the methanolic extract of the stems (March). The MS analysis of spot B4 gave an intense ion at *m*/*z* 283.1 [M–H]^−^. This molecular weight fitted well with one output from the DNP query: 4',5-dihydroxy-7-methoxyisoflavone reported in *Genista carinalis* [21]. One last spot (B5) seemed to exhibit antioxidant activity. Its *R_f_* was slightly higher than the one measured for B4, and the colour obtained after derivatisation with 2-aminoethyldiphenyl borate was different. Unfortunately, the TLC-MS analysis did not give any good results, as several ions with comparable intensities were observed. Surprisingly, several compounds revealed by 2-aminoethyldiphenyl borate (Figure 3a, *Rf* 0.1 to 0.4) did not react with DPPH, despite their phenolic structure.

### 2.1. Identification of Compound **4**

In order to confirm the identification of compound **4**, we proceed to the purification of the crude methanolic extract. The purification yielded 340 mg of compound **4**. A ^1^H-NMR spectrum was acquired and the chemical shifts observed matched those previously published by Mekkio *et al*. The absolute configuration of the sugar moiety was determined by comparison of the sign of the specific rotation with the data published after total asymmetric synthesis of **4** [22]. The positive sign confirmed a D configuration of the glucose. Compound **4** was then applied to the HPTLC plates in track 8 to compare the *R_f_* with the other extracts (B6).

### 2.2. Radical Scavenging Capacity (DPPH)

As sample 1 appeared to us as the most interesting concerning its antioxidant potential, percentage of inhibition of 2,2-diphenyl-1-picrylhydrazyl (DPPH) radical by the crude extract was evaluated. Concentrations from 5 × 10^−3^ mg mL^−1^ to 15 × 10^−3^ mg mL^−1^ were analysed in order to establish the IC_50_ which was calculated as 8.27 µg mL^−1^ corresponding to 0.49 mg of dried plant. The percentage of inhibition of 2,2-diphenyl-1-picrylhydrazyl radical by the purified compound **4** varied from 15.12% (at 20 µg mL^−1^) to 34.43% of inhibition (80 µg mL^−1^), so no IC_50_ was calculated. These results show that compound **4** is a weak contributor to the total radical scavenging capacity of the root extract.

## 3. Experimental

### 3.1. General

Solvents were purchased from Carlo Erba-SDS (Val de Reuil, France). Water was distilled from deionized water. The automatic TLC sampler (ATS4) and the automatic developing chamber (ADC2) from CAMAG (Muttenz, Switzerland) were used for the HPTLC. Detection was realised using the TLC visualiser, the TLC scanner 3 and the TLC-MS interface also purchase from CAMAG. Derivatisation of the plates was performed using the DESAGA sprayer. For TLC-MS, a binary HPLC Agilent pump (Agilent Technologies, Santa Clara, CA, USA) coupled to a ABsciex Qtrap 2000 triple quadrupole mass spectrometer. The mass spectra acquisition was realised using electrospray ionisation in the negative mode. An ARMEN Modular 2 heads pump connected to a Varian Pursuit pre-column and column (C18, 250 × 300 mm) and a Büchi UV/Vis detector was used for preparative HPLC purification.

### 3.2. Plant Material

Different samples of *Genista saharae* Coss. & Dur. were collected at the station of Chott el Hodna (Oued El-Maadher, Boussaâda, Wilaya of M’Sila, Algeria). Specimens were identified by Professor Mohamed Kaabeche (University Ferhat Abbas, Setif, Algeria). Voucher specimens are kept in the Museum of the Department of Pharmacognosy (University Paris-Descartes). Samples were collected in relation with phenologic state of the plant. A first collection was performed during the flowering phase in March 2010 (flowers, stems and roots). A second sampling was performed after the fructification phase in August 2010 (fruits, stems and roots). All samples were dried at room temperature, protected from light. Seeds were then separated from teguments of dried fruits. The seven samples were then crushed and submitted to extraction.

### 3.3. Extraction

Ten g of each sample material were extracted with MeOH (100 mL) at room temperature for 60 min. The solvent was then evaporated under reduced pressure at 40 °C yielding 6.69 to 18.00 g of crude extract. Extraction yields of each sample are presented in Table 1.

### 3.4. Total Polyphenol and Flavonoids Dosage

#### 3.4.1. Total Phenolic Content

Total phenolic content of each sample was determined by the Folin-Ciocalteu method, as described in the European Pharmacopoeia 8th edition [23]. An aliquot of each powdered samples (1.0 g) was extracted with water. Briefly, 5.0 mL of the adjusted solutions (1.0 g in 250 mL) were mixed with water to a total volume of 25.0 mL. To 5.0 mL of the obtained solutions, phosphotungstic reagent (1.0 mL) was added and completed with a calcium carbonate solution (150 g L^−1^ in water) to a total volume of 50 mL. The absorbance at 715 nm (*A* extract) was measured exactly after 2 min after this last addition and compared to the compensation solution. The reference solution of pyrogallol was prepared as described in [24], and the same method was applied to determine the reference absorbance (*A* pyrogallol), at 715 nm. The percentage of total polyphenols, expressed as pyrogallol were calculated according to the following formula:

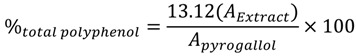



Experiments have been performed in triplicate. Results are shown in Table 2.

#### 3.4.2. Flavonoids Content

The total flavonoids content was determined following the aluminium chloride colorimetric method described in European Pharmacopoeia 8th edition [24]. Briefly, powdered samples (0.25 g) were refluxed in methanol for 30 min. A portion (5.0 mL) of the adjusted methanolic solutions (2.5 g L^−1^) was mixed with an aluminium chloride methanolic solution (20 g L^−1^) to a total volume of 20.0 mL. After 15 min, the absorbance was measured at 420 nm in comparison with a compensation solution. The percentages of total flavonoids, expressed as hyperoside, were calculated according to the following formula, taking the specific absorbance of hyperoside at 420 nm to be 400:
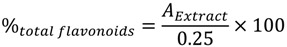



Experiments were performed in triplicate. Results are shown in Table 1.

### 3.5. 2,2-Diphenyl-1-picrylhydrazyl (DPPH) Radical Scavenging Capacity Assay

The DPPH assay was carried out as follows: For sample 1 methanolic extract: A stock solution of sample 1 extract was prepared (16.32 mg of extract in 10 mL, equivalent to 1 mg/mL of dried plant part). An aliquot (0.3, 0.6, 0.9, 1.2 mL, corresponding to a 0.05, 0.10, 0.15, 0.20 mg of dried plant part) of the stock solution was mixed with a methanolic solution of DPPH (1 mM, 1 mL), and brought to 10 mL with methanol. After incubation in the dark at room temperature for 15 min, the absorbance of the solutions was measured at 517 nm. A DPPH blank sample was prepared (1 mL of 1 mM DPPH methanolic solution with 9.0 mL of methanol). The absorbance was readily measured. The decrease in absorbance was recorded for each concentration, in triplicate, and percentage inhibition was calculated according to the following formula:
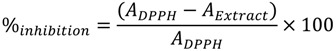



For compound **4**: A stock solution of compound **4** was prepared (31.5 mg in 20 mL of methanol). An aliquot (0.2, 0.4, 0.6, 0.8 mL, corresponding to a 0.315 to 1.25 mg of compound **4**) was mixed with a methanolic solution of DPPH (1 mM, 1 mL), and brought to 10 mL with methanol. After incubation in the dark at room temperature for 15 min, the absorbance of the solutions was measured at 517 nm. The percentage of inhibition was calculated following the above formula. The assay was realized in triplicate. Results are presented in Section 2.2. For sample 1 extract, the plots of the percentage of inhibition in relation of amounts of dried plant parts was used to find the concentration at which 50% radical scavenging occurred (IC_50_).

### 3.6. HPTLC-DPPH Scavenging Assay

The separation of the compounds was carried out using HPTLC silica gel 60 F254 plates 200 × 100 mm obtained from Merck (Darmstadt, Germany) using two mobile phases: ethyl acetate-formic acid-acetic acid-water 100:11:11:26, v/v/v/v and dichloromethane-methanol 95:5, v/v. After development, the solvant was removed heating the plate at 40 °C for 5 min on a CAMAG TLC plate heater. The DPPH scavenging assay was performed by spraying a 0.05% solution of DPPH on the plate to reveal the zones with antioxidant activities. Quercetin (0.1 mg mL^−1^) served as positive control. Two µg of each sample were applied on the plates as follow: roots August (track 1), roots March (track 2); branch August (track 3); branch March (track 4); flowers (track 5); seeds integument (track 6); seeds (track 7); compound **4** (track 8).

### 3.7. HPTLC-MS

For the HPTLC-MS analysis, the interface was connected between the binary pump and the mass spectrometer equipped with the ESI source. An oval shaped extraction head was use for the extraction of the active compounds from the HPTLC plates using a methanol with a flow rate of 0.2 mL min^−1^. Acquisition of the MS spectra was performed in negative ionisation mode with a curtain gas flow rate of 40, temperature of 550 °C and a spray equal to −4,500 V. Most probable *m*/*z* values of the respective zones are listed in the Table 2.

### 3.8. Purification of Compound **4**

Two g of crude methanolic extract of sample 1 (roots, August) was dissolved in a water-methanol 8:2 v/v mixture (10 mL) and injected onto the preparative HPLC apparatus. The column was eluted with a linear gradient of methanol in water (100:0 to 75:25), at 10 mL min^−1^ to give 340 mg of compound **4** was isolated (yield: 17%) as a colorless powder; 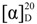 = +17; (c 0.1, CH_3_OH); ESIMS *m/z* 445.2 [M−H]^−^; ^1^H-NMR as reported in [20].

## 4. Conclusions

Whereas the DPPH scavenging assay is widely used for identification of antioxidant compounds, combination with HPTLC-MS and comparison to the DNP database represents an efficient approach. In the case of *Genista saharae* Coss. & Dur. methanolic extracts, both polar and apolar isoflavones were found to be the main contributors to antioxidant activity, whereas other phenolics did not react with DPPH at the tested concentrations. These isoflavones were putatively identified as 3',4',5,7-tetrahydroxyisoflavone (**1**), ficuisoflavone (**3**), 5-methoxy-4',7-trihydroxy-8-glucopyranosylisoflavone (**4**) and 4',5-dihydroxy-7-methoxyisoflavone-4'-*O*-β-d-glucopyranoside (**5**), according to the matching of MS values with *Genista* genus isolated compounds recorded in the DNP database. Confirmation of the structure of 5-methoxy-4',7-trihydroxy-8-glucopyranosylisoflavone (**4**) was in accordance with previous report of this isoflavone in *Genista saharae* Coss. & Dur.

## Supplementary Materials

Supplementary materials can be accessed at: http://www.mdpi.com/1420-3049/19/4/4369/s1.
