# Supplementary material for: Rapid Identification of Antioxidant Compounds of Genista saharae Coss. & Dur. by Combination of DPPH Scavenging Assay and HPTLC-MS
Source: Molecules. 2014 Apr 9;19(4):4369–79. doi: 10.3390/molecules19044369 (PMC6271480; doi:10.3390/molecules19044369)

Supplementary Materials

Figure S1. 5-Methoxy-4',7-trihydroxy-8-glucopyranosylisoflavone (**4**).

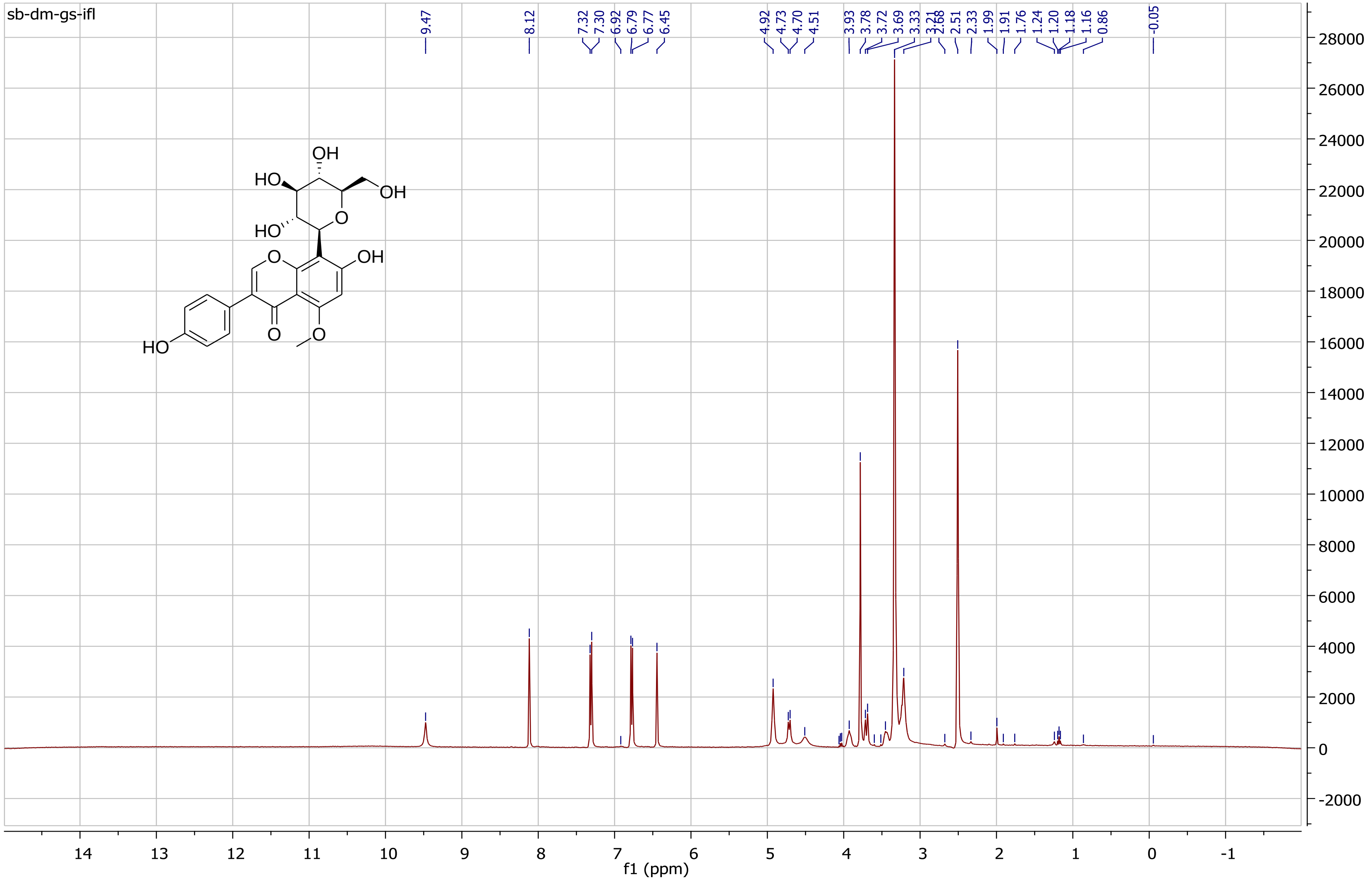

Figure S2. Spot A1.

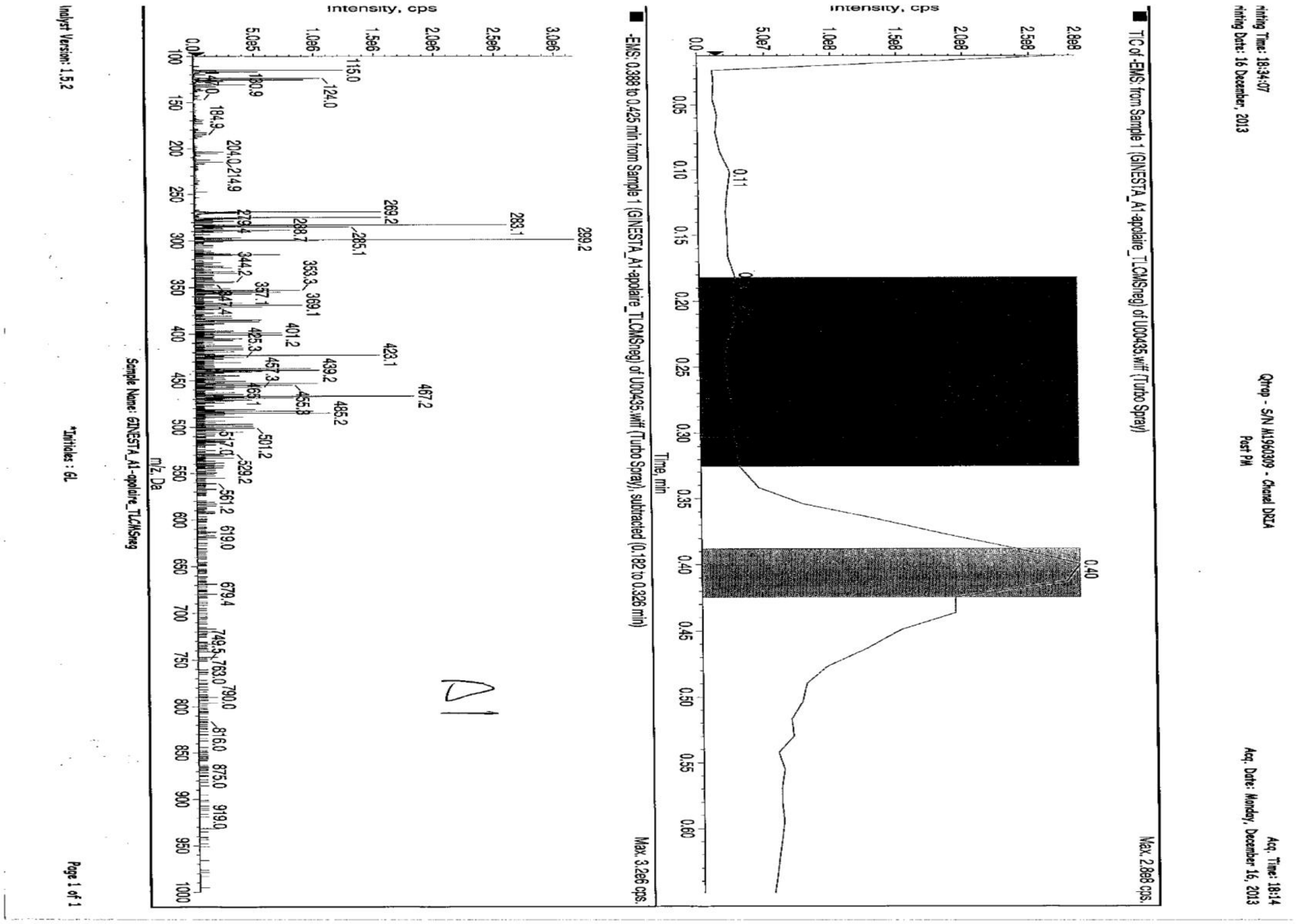

Figure S3. Spont A2 Compound **1**.

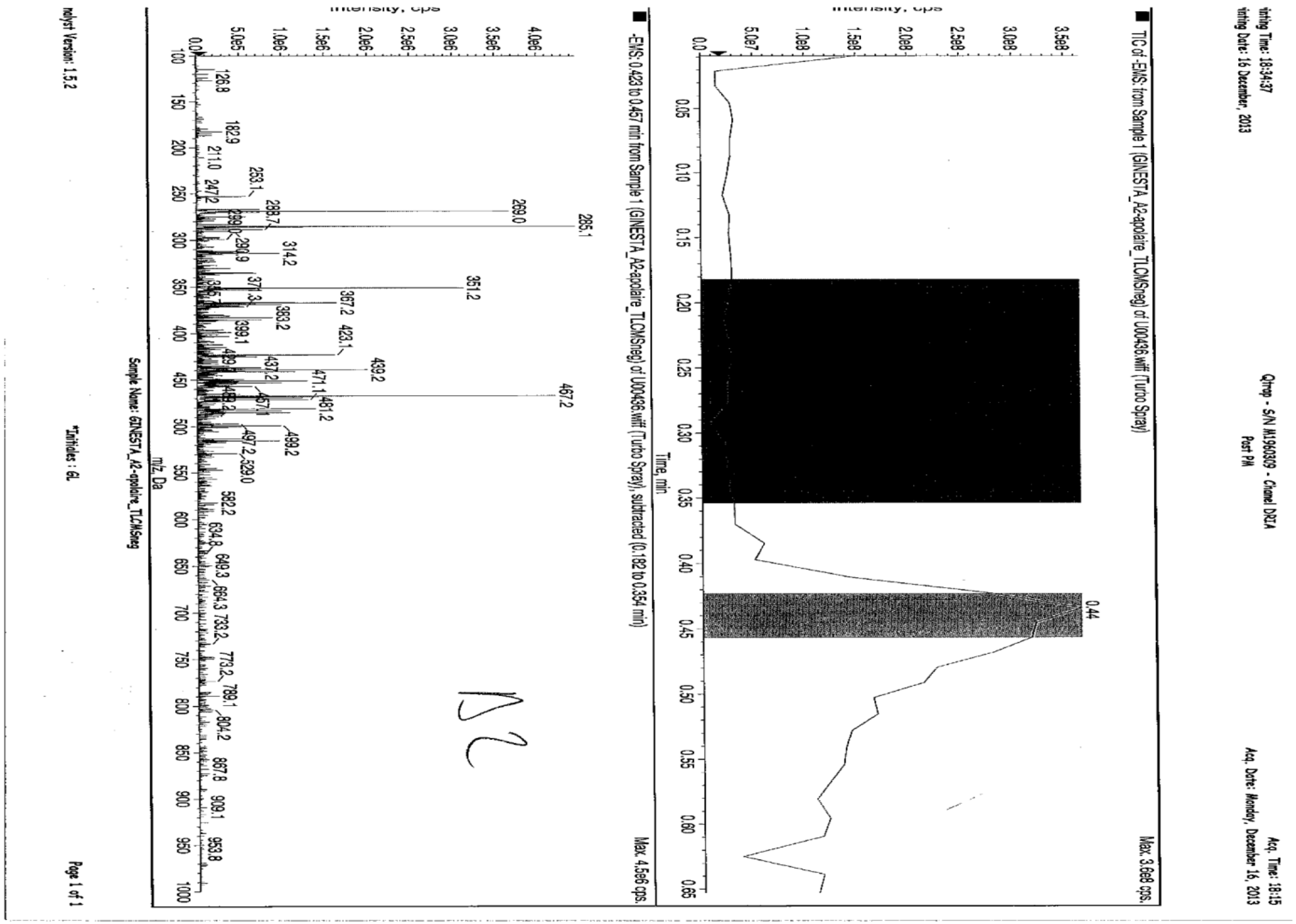

Figure S4. Spont A3 Compound **2**.

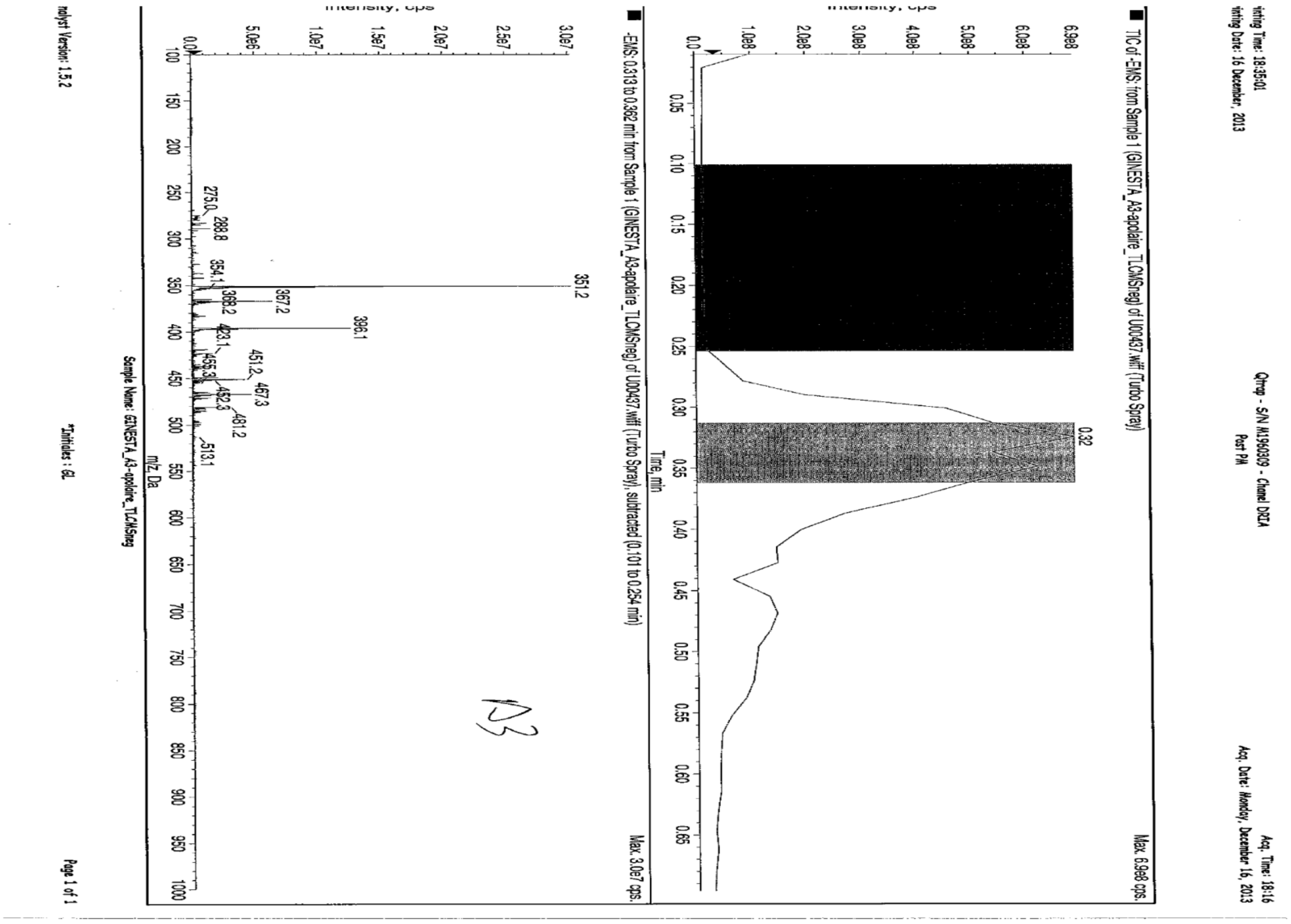

Figure S5. Spont A4.

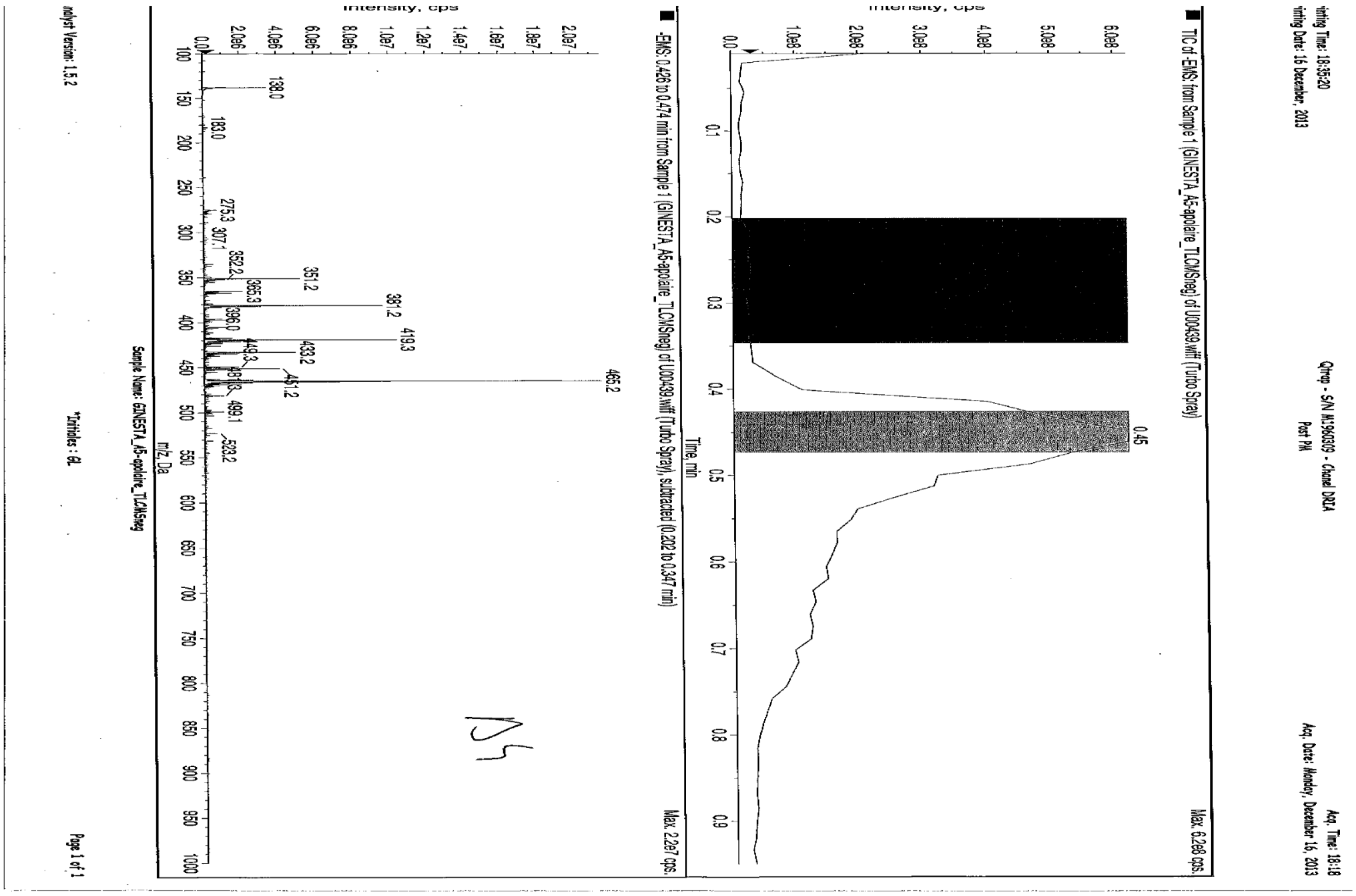

Figure S6. Spont A5 Compound **3**.

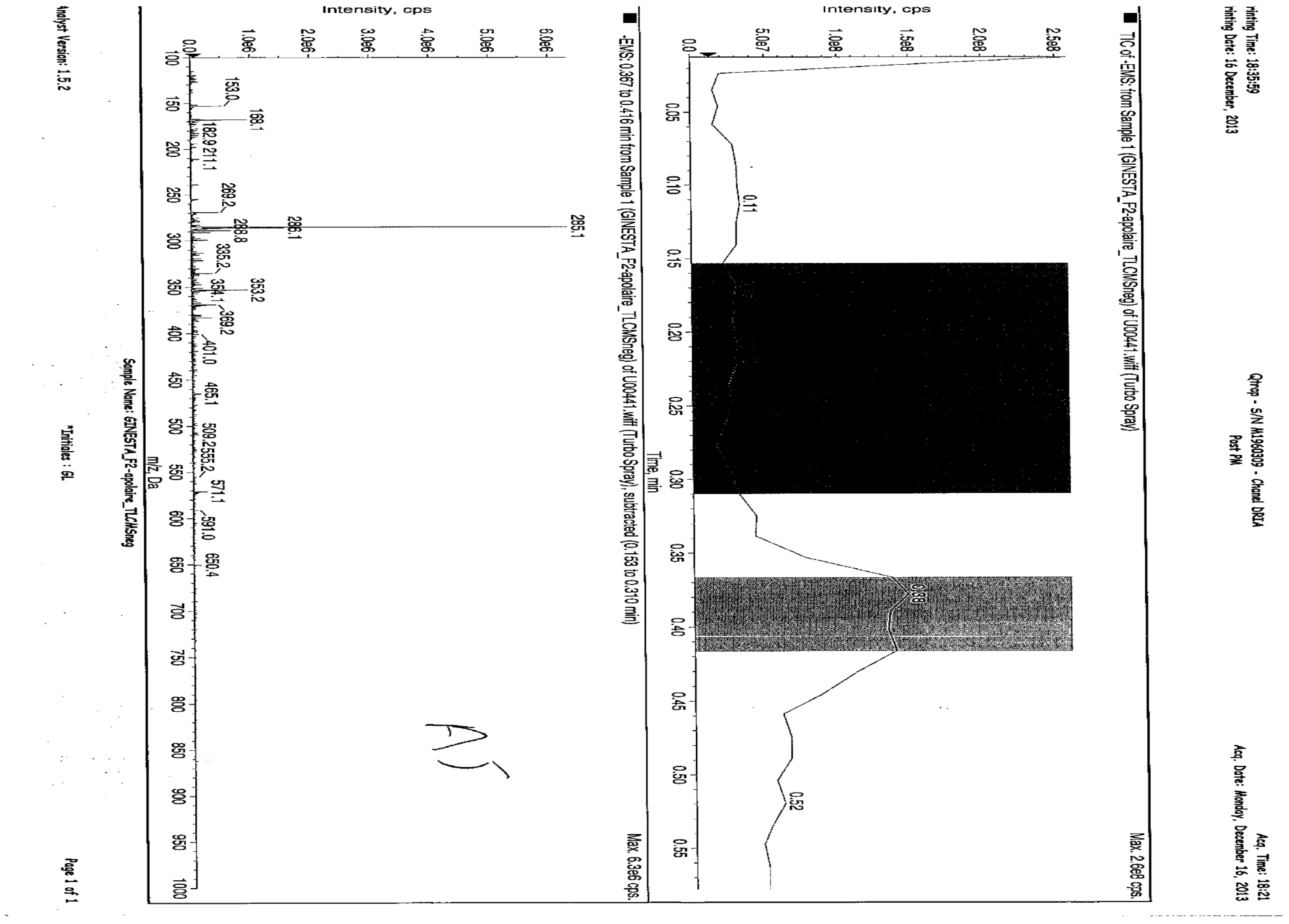

Figure S7. Spont B2 Compound **4**.

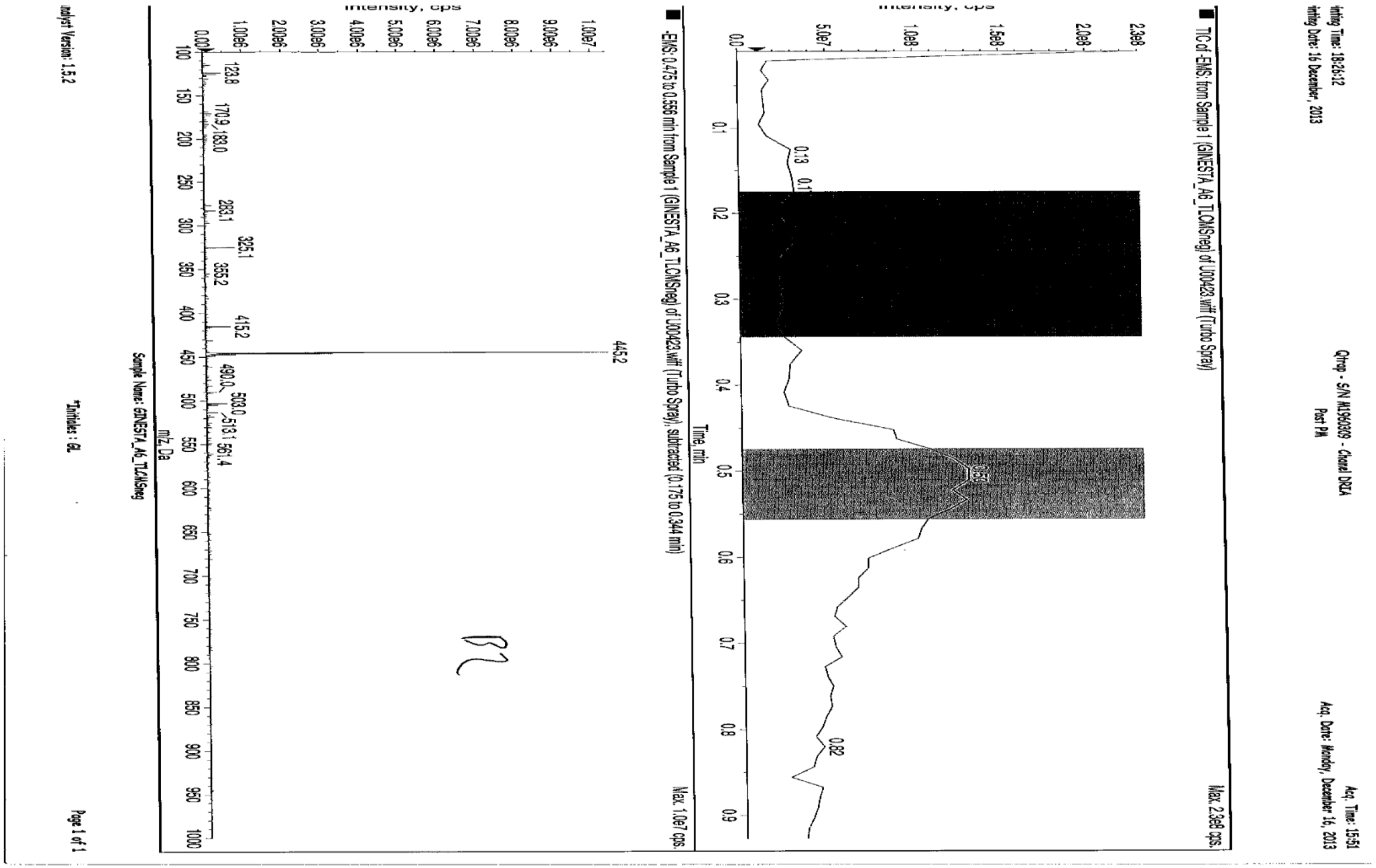

Figure S8. Spont B3 Compound **5**.

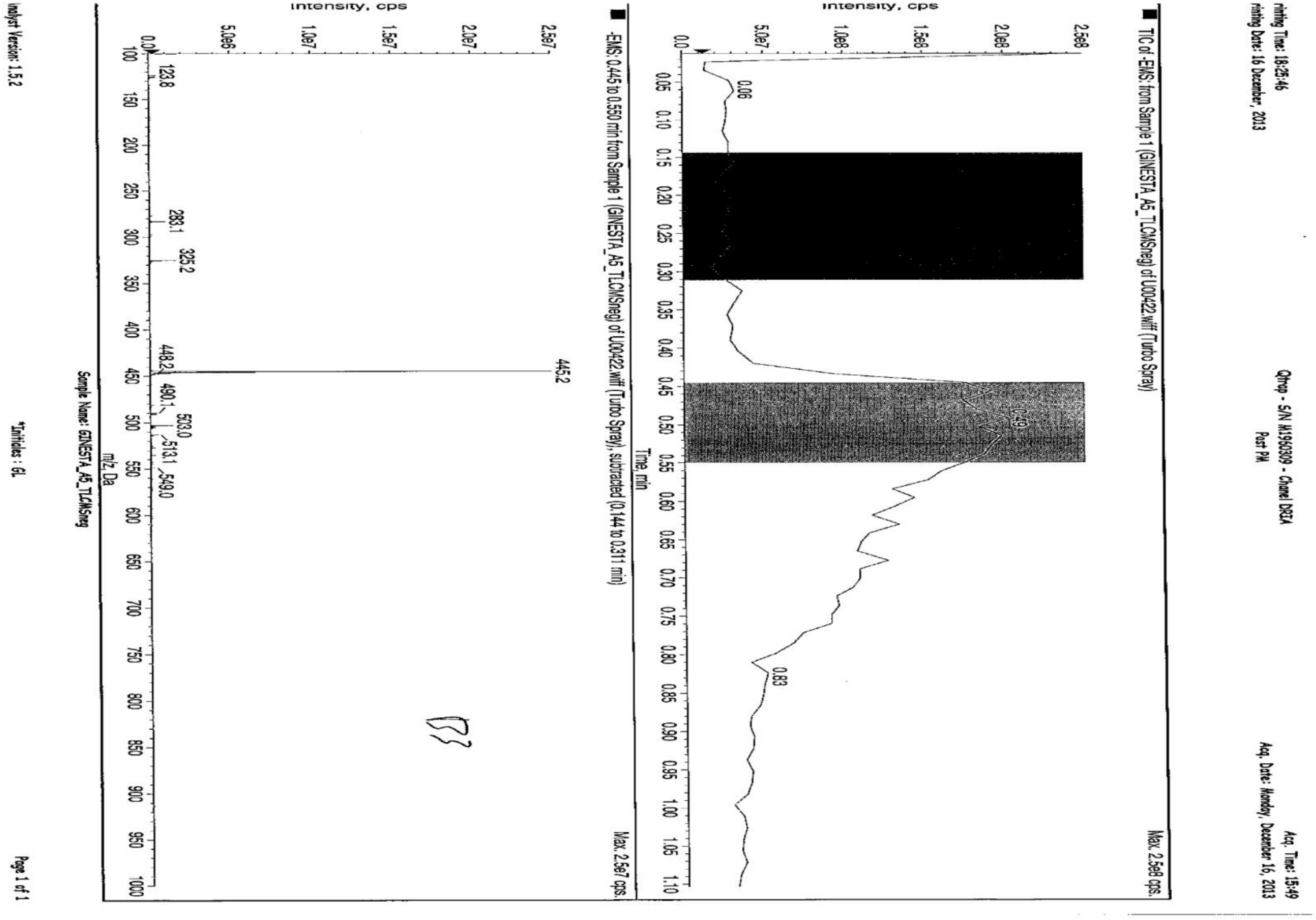

Figure S9. Spont B4 Compound **6**.

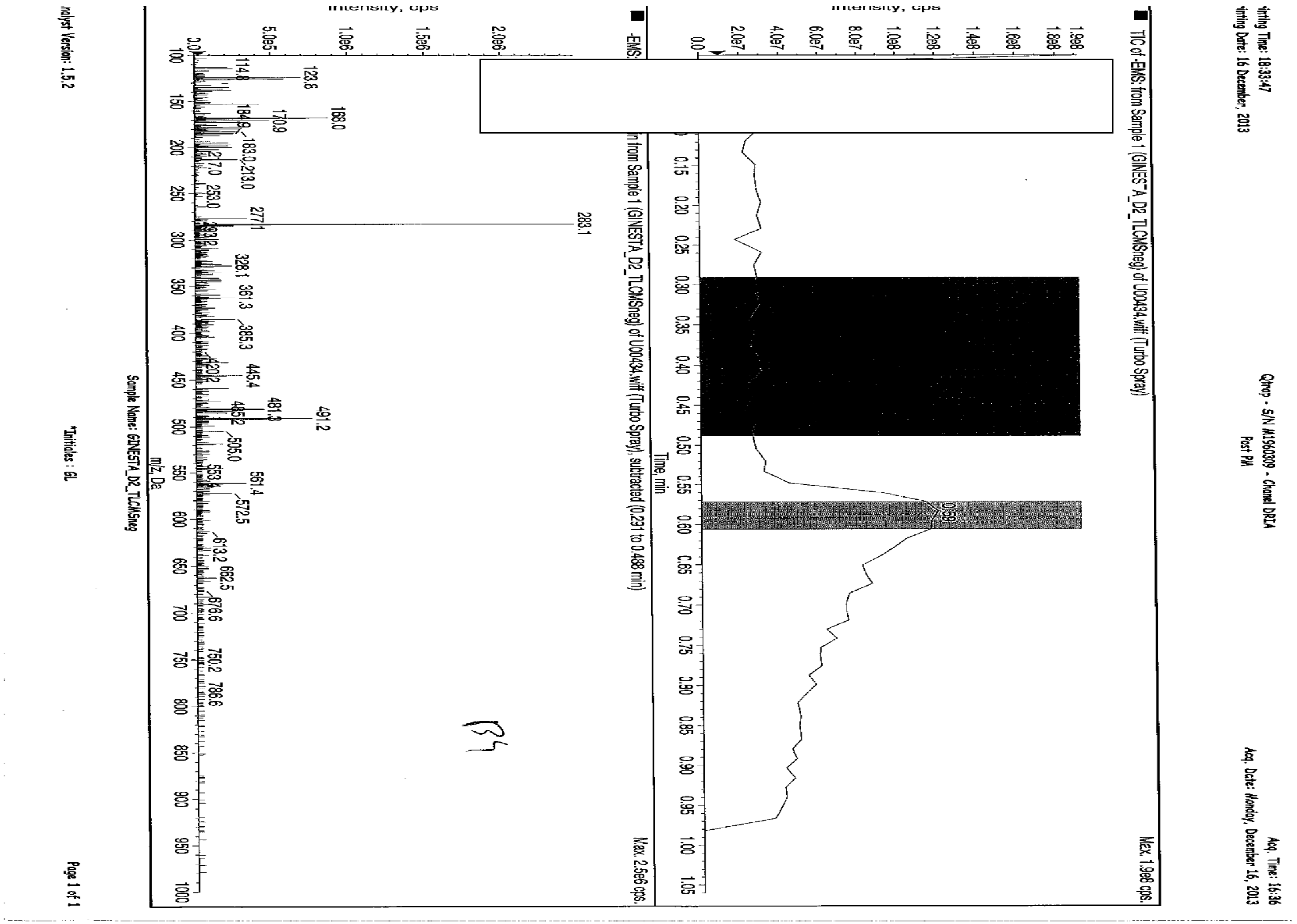

Figure S10. Spont B5.

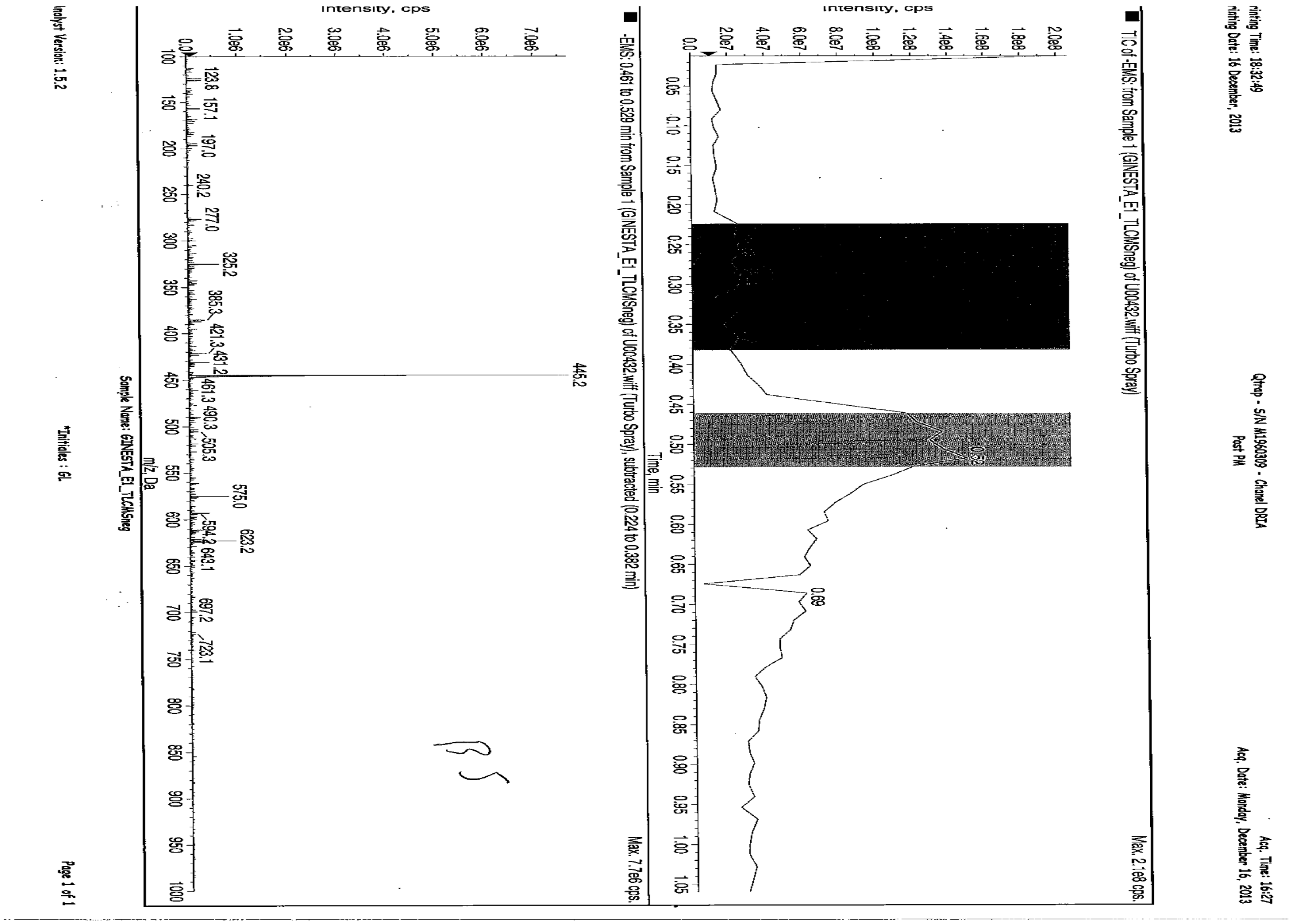

Supplement: Supplementary file 1 [file molecules-19-04369-s001.pdf]
